# Successful treatment of esophagopleural fistula following pulmonary resection for primary lung cancer: a case report

**DOI:** 10.1186/s40792-019-0638-1

**Published:** 2019-05-14

**Authors:** Keisuke Eguchi, Masaharu Ogura, Kazuhiko Hisaoka, Emima Bekku, Shutaro Suda, Tatsuya Shimogawara, Yoshie Kadota, Shigeshi Ono, Fumitaka Asahara, Yutaka Takigawa, Noriaki Wada, Hirotoshi Hasegawa, Junichi Matsui

**Affiliations:** Department of Surgery, Tokyo Dental College Ichikawa General Hospital, 5-11-13 Sugano, Ichikawa, Chiba, 272-8513 Japan

**Keywords:** Esophagopleural fistula, Pulmonary resection, Open-window thoracostomy, Omentopexy

## Abstract

**Background:**

We report a rare case of esophagopleural fistula (EPF) developing during the postoperative period after pulmonary resection for primary lung cancer.

**Case presentation:**

A 71-year-old male who underwent video-assisted thoracoscopic right lower lobectomy with lymph node dissection for primary lung cancer developed severe stabbing pain in his right shoulder and high fever 3 days after the operation. The fever persisted, the cough became more productive, and a plain chest X-ray showed slight a few infiltrative opacities in the right lung field. Intravenous antibiotic therapy was initiated. The patient developed a right pneumothorax 5 days after the operation, and contaminated discharge from the right chest tube was noted. A chest computed tomography showed right-sided empyema, while bronchoscopic examination revealed no evidence of a bronchopleural fistula. Open-window thoracostomy (OWT) was performed. Finally, 2 days after the OWT, the patient was diagnosed as having an EPF, because the right chest cavity was found to be contaminated with food materials. Ample purification of the right chest cavity was achieved by repeated dressing changes, and the EPF was finally closed by omentopexy. The post-surgical course was uneventful. Five weeks after the omentopexy, an esophagogram revealed no leakage of the contrast medium from the esophageal wall. The patient was discharged 13 weeks after the omentopexy.

**Conclusion:**

While EPF following pulmonary resection is a rare complication, it can lead to critical situations and the diagnosis is difficult. Prompt OWT and omentopexy were found to be effective treatment procedures for EPF following lung surgery.

## Background

Esophagopleural fistula (EPF) is a rare but serious complication that can develop after pulmonary resection for primary lung cancer. If EPF occurs in the early postoperative period, it could be difficult to differentiate it from a bronchopleural fistula (BPF) or acute empyema complicating postoperative pneumonia [1]. The clinical course of EPF is often very progressive. Therefore, prompt diagnosis and immediate initiation of appropriate treatment are important. We report a case of EPF that developed following pulmonary resection for primary lung cancer.

## Case presentation

**Fig. 1:**
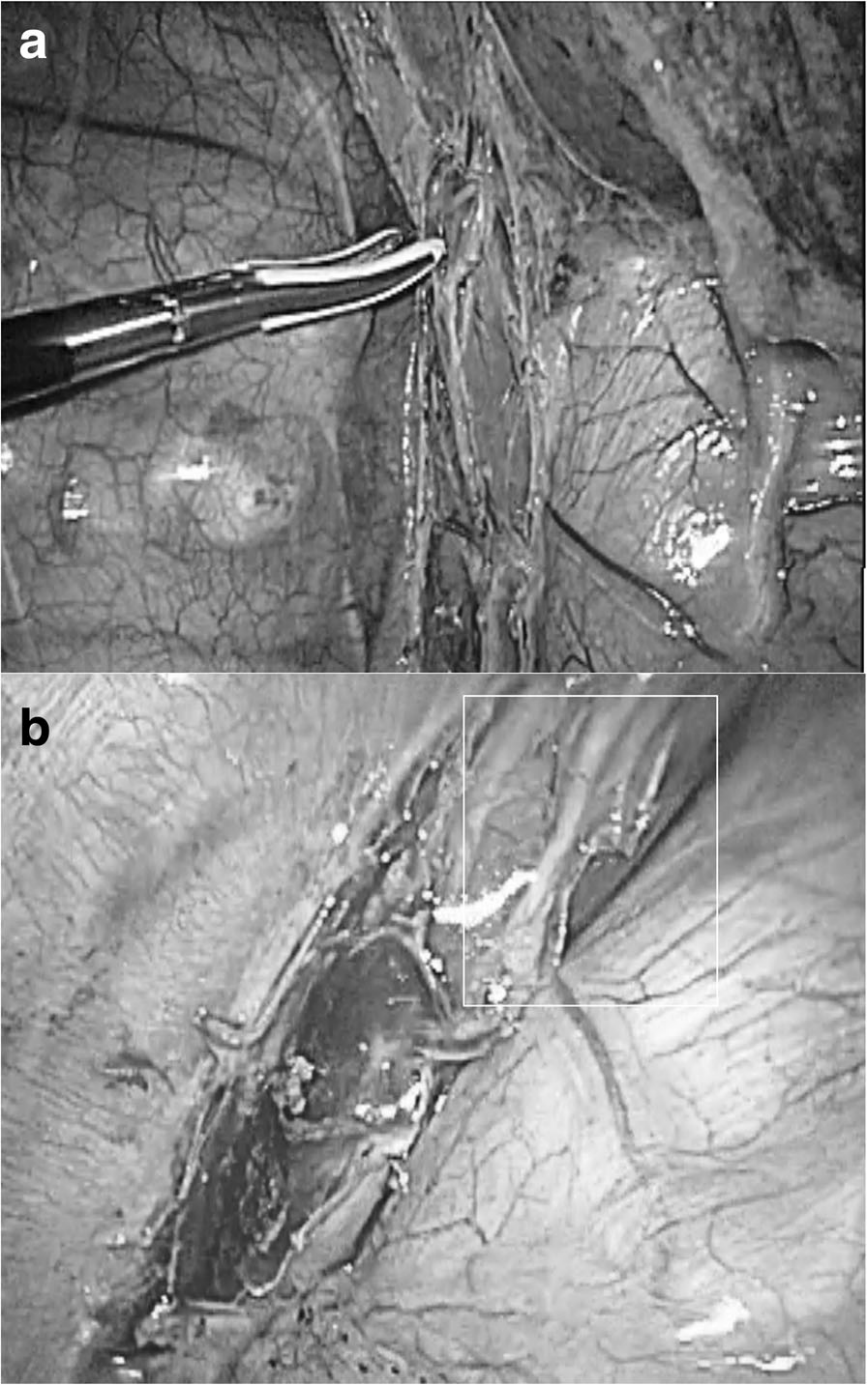
Intraoperative view doing right lower lobectomy. **a** The inferior pulmonary ligament was divided up to the lower border of the inferior vein. No lymph node enlargement was found in the area. **b** No significant damage of the esophagus was identified in the magnified video-endoscopic images obtained after lobectomy (the presumable area where the fistula occurred is shown with a white quadrangle)

A 71-year-old male with a history of alcoholism and radiation treatment (total dose of 57.6 Gy) for early-stage vocal cord cancer 16 months earlier was referred to us with radiographic detection of a pulmonary nodule in the right lower lobe. Endobronchial biopsy of the pulmonary nodule revealed the diagnosis of squamous cell carcinoma. Positron emission tomography/computed tomography (CT) revealed accumulation in the pulmonary nodule, but no other lesion was suggestive of metastasis. The tumor was diagnosed as a primary lung cancer, clinical stage IA1 (T1aN0M0), or metastatic pulmonary tumor, and video-assisted thoracoscopic right lower lobectomy with lymph node dissection was performed. Level 8 and 9 lymph nodes were not enlarged; therefore, lymph node dissection of these nodal station was not performed (Fig. 1a), while the level 7 lymph nodes were dissected. There was no direct injury of the esophagus, and no apparent esophageal damage was visualized on magnified video endoscopic images (Fig. 1b). The operation was uneventful. The patient was able to resume oral intake from the day after the surgery.

The patient tended to need to make a strong effort to cough, with swallowing and expectoration having become difficult, presumably due to the radiation therapy given to the larynx. Three days after the operation, the patient developed severe sudden pain in the right shoulder with high fever (39.6 °C). Findings of the chest X-ray obtained with a portable apparatus showed a few infiltrative shadows in the right lung field, and blood examination revealed no findings that were not compatible with the postoperative status of the patient. The patient was started on intravenous antibiotic administration. Absence of air leakage through the chest tube was confirmed, and the chest tube was removed 4 days after the operation. However, a plain chest X-ray revealed increased infiltrative opacities in the right lung field, and the patient developed a right pneumothorax 5 days after the operation. A chest tube was re-inserted into the right pleural cavity. Sputum mixed with a small amount of blood and cloudiness of the discharge from the right chest tube was confirmed 7 days after the operation. Chest CT showed a marked increase in the size of the right pleural effusion, with air bubbles visualized within the opacity. Although suture failure at the bronchial stump could not be confirmed by bronchoscopy, empyema due to BPF was suspected at first, and open-window thoracostomy (OWT) of the right chest was performed.

**Fig. 2:**
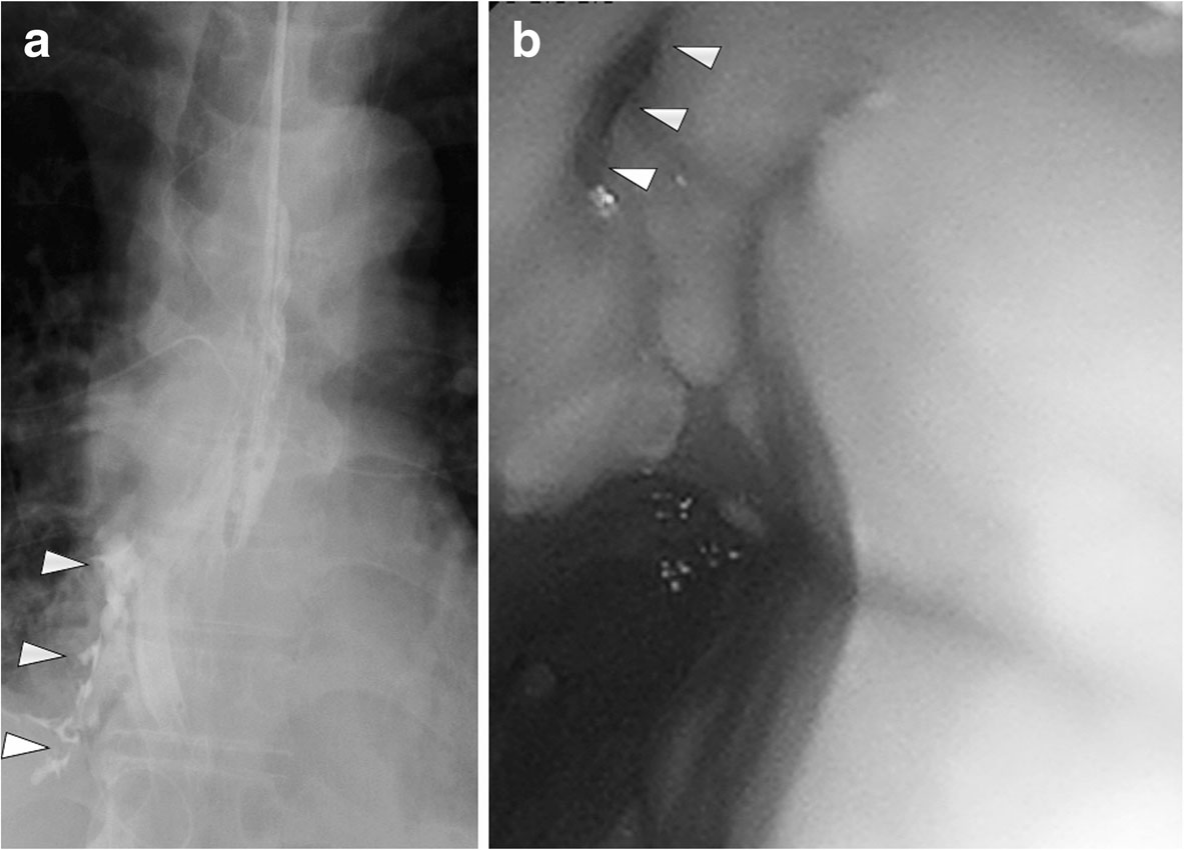
Preoperative imaging findings. **a** An esophagogram showed leakage of the contrast medium from the lower portion of the esophagus into the right thoracic cavity (white arrowheads). **b** Endoscopy showed an orifice of the esophageal wall (white arrowheads)

Two days after the OWT, contamination of the dressing by food particles was confirmed. An esophagogram revealed communication between the lower portion of the esophagus and the right thoracic cavity (Fig. 2a). An upper gastrointestinal endoscopic examination also showed a tiny orifice in the lower portion of the esophagus (Fig. 2b). A nasal W-ED tube™ (Nippon Covidiene Inc., Tokyo, Japan) for simultaneous gastric compression and jejunal alimentation was inserted under endoscopic guidance into the jejunum, and oral intake was totally prohibited.

**Fig. 3:**
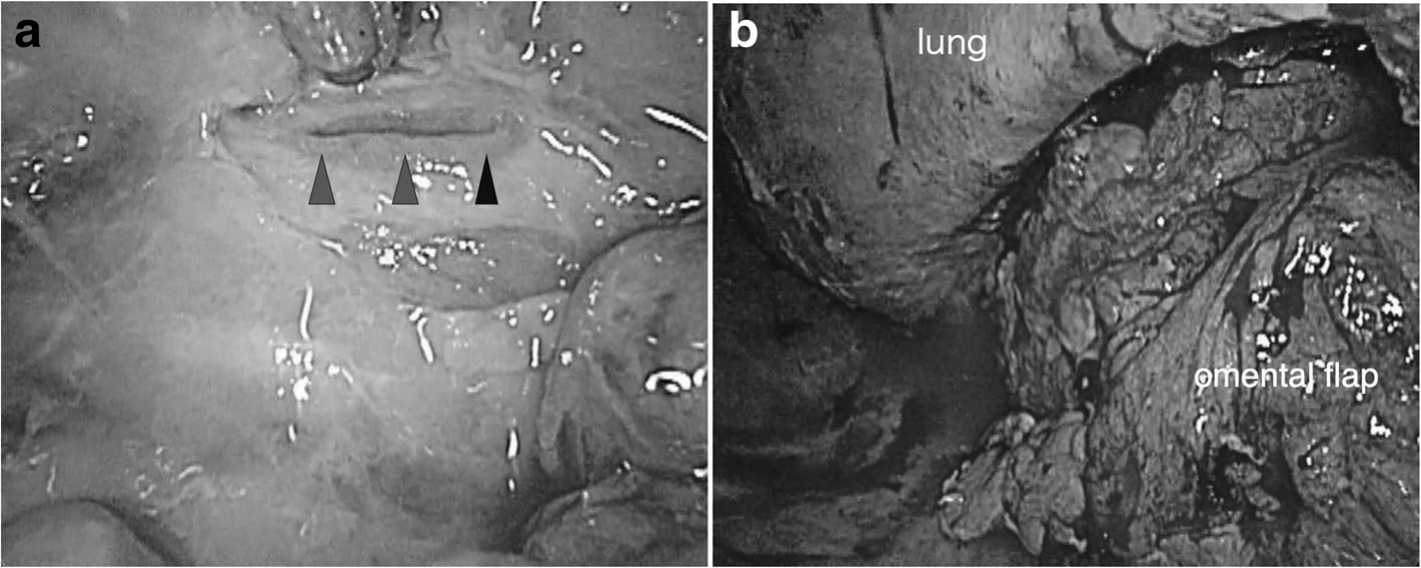
Intraoperative findings. **a** Thoracoscopic findings revealed a small longitudinal tear in the lower portion of the esophagus (black arrowheads). **b** An omental pedicle flap was transferred to the right thoracic cavity through the diaphragm, sutured around the orifice of the esophageal fistula, covering it

**Fig. 4:**
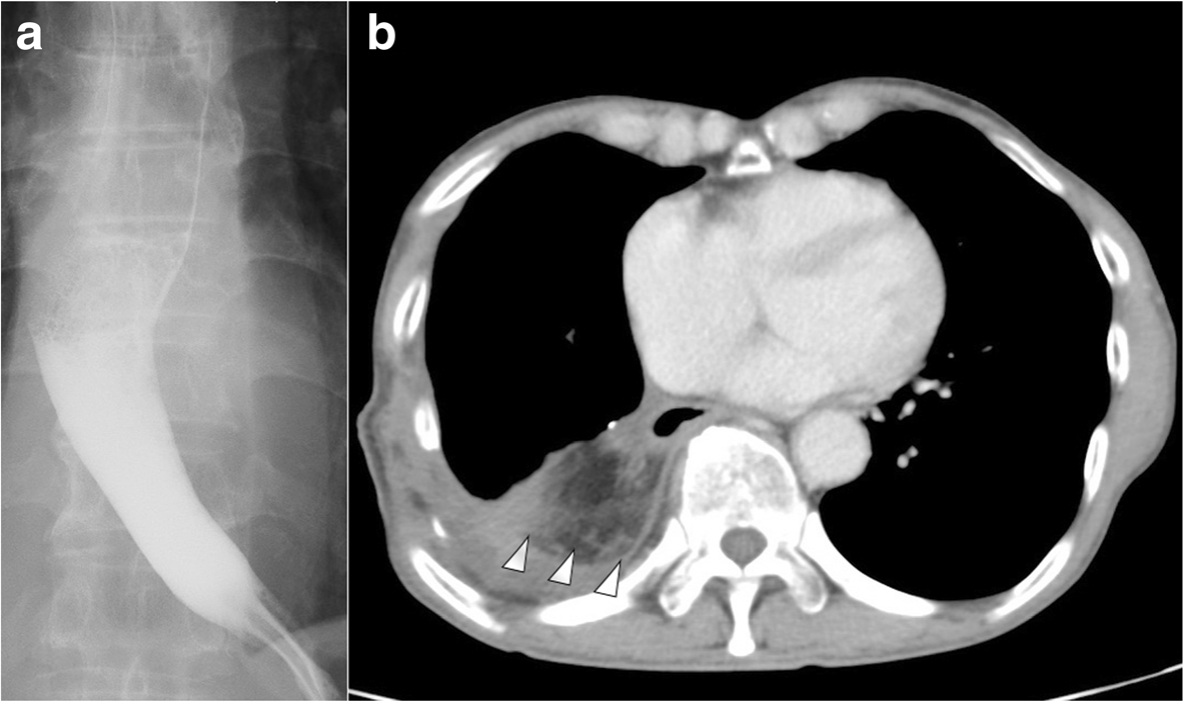
Postoperative image findings. **a** An esophagogram obtained 7 weeks after the operation showed neither leakage of the contrast medium from the esophageal wall, nor stenosis of the esophagus. **b** Chest CT performed 5 months after omentopexy showing the omental pedicle flap filling the surplus pleural space in the right thoracic cavity (white arrowheads)

Dressing changes twice daily were continued, until culture of the thoracic drainage fluid no longer grew pathogens. Omentopexy was performed 20 days after the OWT. A small longitudinal tear, about 1.5 cm in length, was identified in the lower portion of the esophagus (Fig. 3a), below the inferior pulmonary vein stump. No pulmonary fistula formation with esophagus or any BPF was identified. Although the edge of the tear was comparatively clear, the omental pedicle flap (OPF) was sutured around the tear without direct suture, because several days had elapsed after the onset of the esophageal rupture (Fig. 3b). Two thoracic drainage tubes were inserted into the thoracic cavity and the wound was finally closed. The postoperative course was uneventful. An esophagogram obtained 7 weeks after the omentopexy showed no leakage of contrast medium through the esophageal wall (Fig. 4a). The patient was discharged 13 weeks after the operation. A chest CT performed 5 months after the operation revealed complete healing of the EPF and resolution of the empyema of the right thoracic cavity (Fig. 4b).

## Discussion

EPF is a rare but serious complication that can develop after pulmonary resection for lung malignancies. Evans reported an incidence rate of EPF after pneumonectomy of 0.5% [2]. There are no precise data about the incidence of EPF after lobectomy; however, a lower frequency than that after pneumonectomy may be expected. Formation of an EPF soon after pulmonary surgery may be related to direct trauma to the esophagus or to its blood supply during extensive dissection, while that in the later period after surgery may be related to the development of a BPF and empyema following the pulmonary resection [1]. In our present case, no apparent direct injury of the esophagus occurred at the time of the lung surgery, and no damage of the esophagus observed on the magnified video-endoscope images obtained after the lobectomy. The paraesophageal lymph nodes were not enlarged; therefore, lymph node dissection was not performed. Subcarinal lymph node dissection was performed in this case; however, the site of fistula formation was away from the subcarinal area. On the other hand, nutritional insufficiency or spontaneous esophageal perforation, such as in the so-called Boerhaave’s syndrome [3], could also have a role in the development of EPF in this case. The patient had a history of alcoholism and frequent violent cough, which was related to radiation therapy for the tumor in the larynx. Additionally, devascularization of the esophageal wall, associated with the surgical procedure, might have predisposed to the formation of EPF.

There are important things in the treatment of EPF developing after pulmonary resection; to purify the infected pleural space thoroughly and to prevent bronchial stump failure due to infection: OWT was a definitive method to resolve these problems [4, 5]. In addition, decompression of the upper gastrointestinal tract and jejunal alimentation during the fasting period are also important; nasal W-ED tube™, formerly devised as the Peustow-Olander tube, was useful for these purposes and for reducing the discomfort of the patient [6, 7].

Even though the orifice did not seem to be crushed, primary suture was avoided, because it was considered that the EPF was not formed just before the operation. Considering local contamination and the possibility of poor blood circulation, omentopexy, with the expectation of local purification and angiogenesis, is considered as a suitable procedure for the treatment of EPF following pulmonary resection [8]. Using an OPF, empyema with EPF can be closed without thoracoplasty. The point that needs to be reflected upon is that a diagnosis was late, because we were not able to foresee the complications. Chest tube removal should not be done as usual either. Single-stage closure may have been possible if I could confirm esophageal fistula formation earlier.

## Conclusion

We report a rare case of EPF that developed after lobectomy performed for primary lung cancer. Immediate OWT and subsequent omentopexy are useful treatment strategies to prevent secondary development of BPF due to empyema.

## Abbreviations

BPF: Bronchopleural fistula
CT: Computed tomography
EPF: Esophagopleural fistula
OPF: Omental pedicle flap
OWT: Open-window thoracostomy
